# An Examination of Consistency in the Incremental Approach to Willingness to Pay: Evidence Using Societal Values for NHS Dental Services

**DOI:** 10.1177/0272989X21996329

**Published:** 2021-03-18

**Authors:** Katherine Carr, Cam Donaldson, John Wildman, Robert Smith, Christopher R. Vernazza

**Affiliations:** School of Dental Sciences, Newcastle University, Newcastle upon Tyne, UK; Yunus Centre for Social Business and Health, Glasgow Caledonian University, Glasgow, UK; Population Health Sciences Institute, Faculty of Medical Sciences, Newcastle University, Newcastle upon Tyne, UK; School of Health and Related Research, University of Sheffield, Sheffield, UK; School of Dental Sciences, Newcastle University, Newcastle upon Tyne, UK

**Keywords:** dentistry, oral health, protest responses, willingness to pay

## Abstract

**Introduction:**

Willingness to pay (WTP) is used to generate information about value. However, when comparing 2 or more services using standard WTP techniques, the amounts elicited from participants for the services are often similar, even when individuals state a clear preference for one service over another. An incremental approach has been suggested, in which individuals are asked to first rank interventions and provide a WTP value for their lowest-ranked intervention followed by then asking how much more they are willing to pay for their next preferred choice and so on. To date, evaluation of this approach has disregarded protest responses, which may give information on consistency between stated and implicit rankings.

**Methods:**

A representative sample of the English population (*n* = 790) were asked to value 5 dental services adopting a societal perspective, using a payment vehicle of additional household taxation per year. The sample was randomized to either the standard or the incremental approach. Performance for both methods is assessed on discrimination between values for interventions and consistency between implicit and stated ranks. The data analysis is the first to retain protest responses when considering consistency between ranks.

**Results:**

The results indicate that neither approach provides values that discriminate between interventions. Retaining protest responses reveals inconsistencies between the stated and implicit ranks are present in both approaches but much reduced in the incremental approach.

**Conclusion:**

The incremental approach does not improve discrimination between values, yet there is less inconsistency between ranks. The protest responses indicate that objections to giving values to the dental interventions are dependent on a multitude of factors beyond the elicitation process.

Estimating monetary values for health services using contingent valuation (CV) can be a useful tool in estimating the benefits a service can provide to the population questioned. It is often a necessity to aid decision making when market values cannot be observed, as is frequently the case with publicly financed health care.^1^ A stated preference technique frequently used in this context, willingness to pay (WTP), elicits the maximum value participants are willing to pay for specific goods or services. However, eliciting WTP-based preferences for multiple health services competing for limited public funds can be contentious.

When WTP is used to elicit values from members of the public for such competing services, the results often contain inconsistencies known as preference reversals, meaning the rank generated from a simple ordering of interventions (stated or explicit rank) and that generated from elicited WTP values (implicit rank) often do not match.^2,3^ It is difficult to determine the cause of this inconsistency, but it has been linked to confusion regarding the exercise or anchoring on the assumed cost of similar services.^4^ However, these preference reversals could be an indicator that participants are not giving an accurate representation of value, presenting a more fundamental problem for current WTP methodology.^5,6^

Furthermore, WTP exercises often generate nondiscriminatory values (i.e., participants state values with little or no significant difference across services). Depending on the nature of the research and anticipated use of results, this can also be problematic. It is possible that nondiscriminatory values represent true indifference. Alternatively, when options are disparate and it is not unreasonable to anticipate a preference and therefore distinction between values, nondiscriminatory values may indicate participants have not fully engaged with the exercise and may be relying on a behavioral heuristic such as yea-saying.^4^ Results that contain both preference reversals and nondiscriminatory values may indicate the WTP exercise has produced results that may not accurately represent (differences in) value.

An adaptation to the elicitation of WTP was proposed in 2002 to resolve these issues. The “incremental” approach to WTP (originally coined the “marginal” approach) suggested placing an exogenous framework on respondents based on their explicit rank that would eliminate preference reversals and increase discrimination between values.^3^ This approach asks respondents to provide a value for their least preferred option and then asks how much *more* they would pay to attain their next preferred option. This methodology means each value must be as much as or more than the value given before it and therefore eliminates inconsistencies between ranks. Economic theory indicates that by asking how much *more* individuals are willing to pay for each subsequently preferred option, the respondent is encouraged to carefully consider how much additional utility each option provides, forcing individuals to estimate their consumer surplus at each stage, thus encouraging differentiation between options and providing an accurate representation of value.^2^ As respondents are free to give a zero value, meaning an increase in value is not enforced but, provided it is considered a true zero, would indicate the next preference is worth nothing additional.

In contrast, the standard approach usually starts by asking participants to state the order of their preference, followed by asking them to state how much they are willing to pay for each option independently. Previous studies have found the incremental approach to be superior to the standard approach as the former eliminated inconsistencies and increased discrimination between values.^2,7^ Yet, the methodology used in the aforementioned studies discarded protest responses when considering preference reversals. A zero value is considered a protest response if the justification is not related to the intervention itself but an objection to the elicitation process. Provided the participant elicits at least one positive value within the exercise, a zero response in this context is unlikely to be a function of the elicitation system but rather relates to the intervention in question, as an objection to the elicitation system would manifest as protest responses to all interventions. By removing zeros thought to be protests but elicited among other positive values, previous studies have, therefore, eliminated the possibility of identifying inconsistencies in the incremental approach as the framework dictates the value must be the same as, or more than, the previous value in the elicitation process. This analysis considers the role of protest responses and investigates whether they contribute to explicit and implicit rank inconsistencies.

The incremental approach remains relatively unused, as the evidence available with respect to its validity and reliability has been limited since the initial proposal. This article contributes to the body of evidence regarding the incremental approach and presents evidence of its performance relative to the standard approach. The analysis presented evaluates both approaches with respect to preference reversals and discriminatory power in resource allocation for dentistry, a setting that has not yet been explored. The evidence used in this article is from the Resource Allocation in National Health Service (NHS) Dentistry: Recognition of societal Preferences (RAINDROP) study. This study applies priority setting techniques to generate a multicriteria decision making tool—including preferences from the public—to optimize allocation of resources to oral health services in a publicly funded, resource-constrained system. The full protocol for this research can be found elsewhere.^8^ The preference elicitation element of the study establishes monetary values from people residing in England and aggregates them into a societal value for dental interventions. The interventions presented are dental services either currently provided or that have the potential to be provided as part of NHS England dental services or through public health initiatives provided under the umbrella of Public Health England. Previous research using the incremental approach has compared scenarios considered close substitutes, with the most recent evidence examining public preferences for different service providers of a single type of care (emergency and out-of-hours services).^2^ The study described in this article is set in the context of oral health problems faced by the population in England. Preferences are elicited for a broad range of treatments where there is arguably a more complicated tradeoff, with interventions targeting different societal groups.

## Methods

In line with the objectives of the RAINDROP study, a questionnaire was designed to collect WTP values for dental interventions. This was administered by an independent survey company, Qa Research (York, England). Ethical approval was gained from Newcastle University Ethical approval was gained from Newcastle University Ethics Committee (Reference Number 7065/2016).

### Recruitment

Researchers approached households in 50 small local clusters across England, with a maximum of 1 individual recruited in each household. A quota target list was used to ensure recruitment of different demographic groups. Potential participants were given information about the study and gave consent to be interviewed, with a £10 incentive offered. Data collection was undertaken in face-to-face interviews at the interviewee’s home with the interviewer using a computer-assisted interface. Randomization was undertaken at an individual level during the interview as part of the computer-assisted interface to either an incremental or a standard approach using software-based randomization. The algorithm for randomization is built into the survey software used by Qa Research.

### Questionnaire Design

The questionnaire was developed with the aid of a focus group recruited from a Patient and Public Involvement group at Newcastle University to ensure that the questionnaire was understandable and engaging for members of the general public. The questionnaire was then piloted with a small sample of the general public (recruited in the same way as for the main data collection), and small changes to the wording were made.

The questionnaire included 3 distinct sections: the initial section gathered information regarding the participant’s demographics and socioeconomic status. The second section introduced participants to the interventions and collected explicit ranks and WTP values. The third section asked for information on participants’ incomes, their experience of dental interventions, and frequency of dental visits, all of which are hypothesized to affect their maximum willingness to pay for interventions.

#### Explicit ranking

The 5 interventions valued were chosen as part of a workshop with NHS England dental commissioners and clinical dental leaders as areas of interest for potential investment or disinvestment in the NHS. Table 1 offers a brief summary of each intervention.

**Table 1:** Brief Summary of Interventions^a^

Intervention	Problem	Treatment	Population Targeted	Outcome	Number of Individuals Expected to Receive Treatment
Fluoride varnish	Dental decay can cause need for fillings, crowns, or extraction.	Preventive Clear varnish applied to teeth at nursery by a dental nurse.	Children aged 3–5 years, mostly from deprived areas.	Reduces the number of teeth affected by decay (by 37% in 3 years).	216,000
Molar root canal	Back teeth dying off due to deep decay causing an abscess.	Drilling and filling of tooth, placement of crown over 3 appointments.	Adults, prevalence linked to deprivation.	Eighty percent success rate, pain alleviated, the tooth saved. Tooth removed if unsuccessful.	600,000
Extended orthodontics	Misalignment of the teeth.	Aesthetic. Braces placed on teeth and then adjusted every 6 to 8 weeks for 18 months.	Treatment usually at 12 years old; all groups equally likely to need treatment.	Teeth are no longer misaligned.	16 million
Supervised tooth brushing	Dental decay can cause need for fillings, crowns, or extraction.	Preventive Primary school staff trained by dental staff to supervise brushing.	Program aimed at the deprived primary schools in the United Kingdom. Target children aged 3 to 11 years.	Reduces the number of teeth affected by decay between 10% and 40%.	800,000
Care home visits	Residents require dentures but cannot attend a dentist.	Dental exams, molds of teeth, and denture fittings taking place in care homes.	Care homes are predominantly female; tooth loss is associated with deprivation.	Affected residents find it easier to eat and talk unassisted, feel more confident showing teeth.	3350–83,750

a The figure associated with care home visits is a range. This is because dentures have a finite life span, with a replacement rate of approximately once every 5 years, meaning a maximum of 83,750 and a minimum of 3350 are estimated to need replacing in a care home setting per year.

Respondents were presented with a long-form explanation of each intervention and were asked if they understood each description. For those who did not, a flash card with a succinct version of the key information was presented. These were accessible for each participant throughout the exercise. The order in which the interventions were presented to respondents was randomized to control for ordering and anchoring effects.^9,10^ After respondents had read descriptions for all interventions, they were asked to rank interventions from their most preferred to their least preferred. This will be referred to as their explicit rank and represents an ordinal ranking of alternatives. For this part of the exercise, equal ranking of options was not possible, thereby replicating difficult resource allocation decisions where options are mutually exclusive and the budget is finite.^2^

#### Value elicitation

Before eliciting monetary values, interviewers read a “cheap talk script” to respondents based on Mahieu et al.^11^ This reinforces the hypothetical nature of the exercise and attempts to reduce hypothetical bias by informing the respondents of behavior that is common in contingent valuation settings but not necessarily replicated in real-life situations. The statement was also included to encourage participants to express the value they hold for the intervention, instead of focusing on cost, and to minimize gaming from respondents.

The payment vehicle of extra taxation for the household was chosen for a multitude of reasons. First, most English residents who see a dentist receive their dental care from the NHS.^12^ The NHS is funded through taxation, but in dentistry, there is also a copayment for most users. Second, the interventions discussed are provided at a national level and therefore may not have a direct impact on the household. Taxation encourages an individual value for a service provided at a societal level. Third, the service would be continual, provided year on year for the foreseeable future. Finally, additional taxation fits the wider scope of the project as the RAINDROP study addresses how to best allocate resources within NHS dentistry while taking into account societal preferences. Those who do not pay tax were asked to give an estimation of the maximum amount they would be prepared to voluntarily contribute each year.^13^

Participants were randomized into 2 groups for the value elicitation portion of the exercise, each using a different approach to WTP: standard or incremental. As described previously, the standard approach asks for an absolute, stand-alone maximum value from respondents for each intervention. To ensure the greatest degree of comparability between the 2 approaches, values for the standard approach were collected from the respondent’s least to most preferred option. The incremental approach is a sequential valuation exercise whereby values are elicited by asking participants the maximum they are willing to pay for their least preferred option, then how much more they are willing to pay for their next preferred option. The value of the intervention is therefore partially dependent on the value elicited directly before it, generating an exogenous framework determined by the explicit rank where each subsequent value is equal to or more than the value that came before. For both approaches, a ranking is inferred from the monetary values given for the interventions, with the highest valued intervention corresponding to the most preferred option and the lowest valued option corresponding to the least preferred option. This rank is referred to as the implicit rank.

To elicit the values, respondents were presented with a series of randomized payment cards on their computer interface to avoid anchoring.^10^ The values range from £1 to £200 in various increments and were informed by discussions with the focus group. Respondents had to sort the payment cards into 3 categories: “yes—willing to pay,”“no—not willing to pay,” or “not sure.” Values that were sorted in the “not sure” category were presented again once all other values for that intervention had been sorted, to check if the respondent wished to move any “not sure” cards into “yes” or “no” categories. As the list of payment cards was not exhaustive, respondents were asked after the sorting exercise if they were able to give an exact estimate of the maximum they would be willing to pay using an open-ended question. This gave participants an opportunity to reassess or confirm their valuation. If an exact estimate was not given but there was a response to the payment card portion, a midpoint between the last “yes—willing to pay” and first “no—not willing to pay” was used.^14^ Respondents were able to give a zero response at any point during the exercise.

#### Zero values

When a zero value was given, the respondent was prompted to justify their answer. This justification is used to determine if the response was a true zero or protest response, where a true zero is thought to be an accurate representation of value. The zero justification section used in the questionnaire is largely based on sections developed by Dixon and Shackley^15^ and Ryan et al.,^16^ which used set-text responses and included an open-ended option. Free speech responses to the zero value classification question were transcribed by the interviewer issuing the questionnaire. These were reviewed independently by 2 members of the research team for classification into protest or true zero, with disagreements resolved by discussion.

### Data Analysis

Descriptive statistics are provided for the whole sample. As this article is mainly concerned with the elimination of preference reversal in the incremental approach, the main body of analysis examines this, but the other major purported advantage of the incremental approach, increased discrimination between competing options, is also considered.

#### Preference reversals

To generate evidence regarding preference reversals, responses are sorted into 3 categories: consistent, partially consistent, and inconsistent. These are defined as follows:

- Fully consistent—where the implicit and explicit rank correspond exactly
- Partially consistent—where the deviations between the implicit and explicit rank are due to equal values. For example, if the fifth and fourth preferences both receive the lowest value, but the values increase for the third, second, and first preferences, this is a partially consistent response.
- Inconsistent (preference reversal)—where the implicit and explicit ranks are directly contradictory and the deviation is not due to equal values. For example, if the third preference is valued lower than the fourth preference.

Although partially consistent responses are possible in the incremental approach, the framework placed on respondent valuations for the incremental approach in conjunction with the elimination of protest responses means that preference reversals are impossible. However, for the purposes of this article, it is assumed that, after giving some positive values, a respondent in the incremental approach group then registers a protest response, and this is evidence of an inconsistency between implicit and explicit ranks and thus a preference reversal. This article therefore presents new evidence regarding preference reversals in the incremental approach by retaining all protest responses for the analysis of respondent consistency.

#### Discrimination between values

To examine the discrimination between values for both approaches, we give the mean, standard deviation of the mean, and median associated with each intervention and rank. For the analysis regarding discrimination, responses considered protest responses are discarded, as they are not considered a representation of value and may therefore deflate the societal value unjustly.^13,15,17^ The median values are the main focus of discussion as means are more susceptible to bias from outliers (often driven by high-income respondents who may place high bids on their preferred interventions).

## Results

A total of 790 participants were recruited; 6 participants with incomplete data collection were dropped. The remaining participants were randomized to either the incremental approach (*n* = 335) or the standard approach (*n* = 449). Descriptive statistics for both samples are presented in Table 2.

**Table 2:** Descriptive Statistics

Characteristic	Incremental (*n* = 335)^a^	Standard (*n* = 449)^a^
Sex, male, %	47.46	51.45
Age, %		
16–24	13.73	18.93
25–34	18.51	18.49
35–44	21.79	15.14
45–54	15.52	16.93
55–64	14.63	12.92
65+	15.82	17.59
Median income, 2016 pound sterling (£)	23,400	18,200
Index of multiple deprivation, %		
30% most deprived	41.49	39.87
30% least deprived	13.13	11.8
NS-SEC, %		
1	27.46	29.18
2	12.84	8.19
3	12.84	10.02
4	7.46	8.46
5	22.69	23.16
Other	16.72	20.27
Experience, %		
Fluoride varnish	7.16	7.80
Root canal	36.12	35.41
Orthodontics	26.87	29.18
Supervised tooth brushing	8.36	6.46
Care home visits	1.19	1.11
Opinion on government welfare provision, %		
Too much welfare	27.46	28.51
Right level of welfare	72.54	71.49
Frequency of dental visits, %		
Less than once every 2 years	24.18	25.17
At least once every 2 years	75.82	74.83
Teeth, %		
Fewer than 10	6.27	9.13
10–19	8.36	10.47
20+	85.37	80.40
Experience of dental pain, %		
None	29.81	35.49
Some	67.95	62.83
Current pain	2.24	1.68
Treatment provider, %		
NHS	57.61	57.02
Private	24.78	19.38
Other/don’t know	17.61	23.61

NHS, National Health Service; NS-SEC, National Statistics Socio-Economic Classification.

a The random allocation process produced uneven sample sizes. The survey company used, Qa Research, cannot disclose its algorithm but has assured the research team that the process was random and could not be influenced by the administrators.

The median household income for the sample is lower than the national average for the United Kingdom (£27,300).^18^ All other demographics reported were broadly representative of the United Kingdom for the year 2016, when the data were collected.

### Explicit Rank

The explicit rank is shown in Table 3. For both the incremental and standard approach, the least preferred intervention is providing dentures in care homes. Supervised tooth brushing is most frequently picked as first and second preferences across approaches. Out of 120 possible combinations of intervention ordering with respect to explicit preferences, 115 are present, with the most frequently observed combination only occurring 3% of the time.

**Table 3:** Explicit Preference Ranks

	Rank, *n* (%)				
Intervention	5 (Least Preferred)	4	3	2	1 (Most Preferred)
Fluoride varnish	145 (18.49)	157 (20.03)	126 (16.07)	176 (22.45)	180 (22.96)
Root canal	136 (17.35)	233 (29.72)	168 (21.43)	119 (15.18)	128 (16.33)
Extended orthodontics	165 (21.05)	171 (21.81)	210 (26.79)	152 (19.39)	86 (10.97)
Supervised brushing	144 (18.37)	110 (14.03)	113 (14.41)	180 (22.96)	237 (30.23)
Dentures in care homes	194 (24.74)	113 (14.41)	167 (21.30)	157 (20.03)	153 (19.52)

### Values Elicited

Values for specific interventions and with respect to rank can be found in Table 4. For the incremental approach, median values range from £25 for fluoride varnish to £30 for orthodontics, supervised tooth brushing, and dentures in care homes. The mean values range from £60.77 for orthodontics to £77.95 for supervised tooth brushing.

**Table 4:** Values with Respect to Intervention and Rank in 2016 pound sterling (£)

	Incremental Approach^a^					Standard Approach^a^				
Intervention	Mean	Median	Maximum	SD	*n*	Mean	Median	Maximum	SD	*n*
Fluoride Varnish	69.09	25.00	750	115.66	296	30.59	12.00	300	45.54	391
Root canal	63.98	27.50	1000	114.79	303	33.14	20.00	250	42.76	406
Orthodontics	60.77	30.00	1000	101.07	299	35.53	20.00	400	49.70	400
Supervised brushing	77.95	30.00	800	116.90	288	30.36	10.00	300	46.53	403
Dentures in care homes	69.59	30.00	950	119.65	301	32.80	20.00	220	43.58	402
Rank										
5	20.79	10.00	200	33.68	285	28.72	10.00	200	42.14	381
4	40.84	20.00	400	63.21	296	27.31	10.00	400	41.58	393
3	65.87	30.50	600	96.46	296	30.21	15.00	200	39.79	403
2	89.30	44.50	800	128.53	301	35.20	19.75	250	47.60	412
1	119.79	65.00	1000	163.31	309	40.41	20.00	300	54.12	413

a As the sample sizes were uneven, a decomposition test in the style of Oxaca-Blinder was used to assess if the sample composition was responsible for the difference in value at the mean for the fifth preference. The test indicated that if the groups were randomized to the other approach, they would produce similar results, which are available on request.

In the standard approach, values are lower than in the incremental approach, with the means, maxima, and medians all occurring at lower values. The median values range from £10 for supervised tooth brushing to £20 for root canals, orthodontics, and dentures in care homes. The standard deviations associated with the incremental approach are higher for all interventions. The mean values range from £42.76 for root canals to £49.70 for orthodontics.

### Protest Responses

A total of 423 protest responses were given by 135 participants, the details of which are displayed in Table 5. Only 21 (2.68%) participants across the approaches gave protest responses to each intervention in the valuation process. Generally, the number of protest responses given falls as the exercise progresses. The most frequently objected to intervention for the incremental approach is supervised tooth brushing, while for the standard approach, it is fluoride varnish.

**Table 5:** Protest Responses

	Rank, *n*					
Intervention	5	4	3	2	1	Total
Fluoride varnish	27	25	17	18	10	97
Root canal	16	20	21	10	7	74
Extended orthodontics	24	13	17	24	6	84
Supervised brushing	27	18	15	10	20	90
Dentures in care homes	23	15	14	9	17	78
**Total**	117	91	84	71	60	

### Preference Reversals

A novel approach in this analysis is identifying how protest responses affect consistency of the implicit and explicit ranks. Comparing the approaches, we find that for the incremental approach, 72% (*n* = 228) of respondents were fully consistent, compared with only 2% (*n* = 10) of respondents in the standard approach. The proportions of inconsistent respondents are 11% and 72%, respectively, almost a mirror image of results. The remaining respondents are partially consistent. Examining the sources of inconsistency revealed 2 patterns of protest behavior across approaches:

1. Participants give protest responses at the start of the exercise with the participant joining with positive values later. For example, they may give protest zeros for their fifth and fourth preferences but positive values for their third, second, and first.
2. Participants protest midway through the exercise with positive values occurring after the protest response. It may be that the participant gives a positive value for their fifth preference and a protest zero for their fourth, then continues to give positive values for the remaining 3 interventions.

These are explored further in the discussion.

## Discussion

This study is the first to find preference reversals in the incremental approach. However, the proportion of participants eliciting an inconsistent response in the incremental approach is 11%, compared to 72% in the standard approach. The presence of preference reversals is only problematic insofar as it is believed that there should be complete correspondence between the explicit and implicit rank. This rests on the assumption that the individual responding to the questionnaire is valuing the same attributes in both ranking exercises, yet it is not uncommon to observe individuals providing different values for the same service dependent on perspective in the context of health.^19^ It is therefore possible that, when asked to explicitly rank, individuals place the interventions in order of what they would personally prefer to be funded (i.e., what would have the largest impact on themselves), while the valuation exercise, in asking for additional taxation payments, may encourage a wider perspective. This fluid perspective can be seen in the wider willingness to pay literature. Evidence from the EuroWill study noted that the explicit ranking task and the valuation exercise are fundamentally different.^20^ The explicit rank is an ordinal rank resting on a direct comparison of competing programs, while the valuation exercise that generates the implicit rank changes the frame of reference and asks people what amount they would be willing to sacrifice from their personal income to have the program offered in the public domain. It is therefore important to consider the nature of the 2 ranking exercises and the changing frame of reference when discussing inconsistences in willingness to pay exercises, particularly regarding the implications of inconsistences on validity.

It is also possible that respondents are revising their initial rank, or only arrive at their final rank, as the exercise progresses. The standard approach gives an easy avenue to revisit and revise rank, as values can be assigned freely. The respondents in the incremental approach are somewhat anchored to their initial response as the framework dictates the value must be as much as, or more than, the preceding value, which could be problematic if ranking preferences across disparate bundles is difficult. However, a previous willingness-to-pay exercise has explored the discrepancy between explicit and implicit rank with the respondents, with the majority (75%) of respondents identifying that their explicit ranks should be used to inform priorities in a health service.^20^ If this holds for other priority setting in health exercises, the incremental approach may in fact enable respondents to perform more rationally; the framework may displace heuristics like anchoring on price and allow respondents to more accurately reflect their underlying preferences.

The inconsistent behavior of those who give 1 or more protest responses suggests the unobserved underlying objection may be a result of an amalgamation of factors not strictly associated with the elicitation process, arguably indicating that participants are only willing to support some interventions funded by the specified payment vehicle. These patterns of protest responses indicate participants opt in and out of the exercise dependent on the intervention and surrounding context, as opposed to the exercise itself. The current methodology in determining whether a zero is true or protest is therefore limited, and using traditional methods to determine the true intent behind a zero value is insufficient to decipher a response that is a protest to the elicitation system compared with other, more-specific objections regarding the interventions and payment vehicle. This is shown by the inconsistent protest behavior in our sample. Using the traditional approaches limits insight into the nature of the protest and potentially limits the representativeness of the sample.^21,22^

The values associated with the interventions offer little discrimination at the whole sample level for either approach, and both suffer from significant clustering at the median, which could suggest a true lack of differentiation in strength of preference or a lack of sensitivity in both approaches. When considering a true lack of differentiation, this may be due to the disparate nature of alternatives and the resulting spread of preference among options. In previous studies regarding the incremental approach, there has been a clear consensus rank between options, which potentially has contributed toward discrimination between interventions.^2^ As there is no common rank, and the incremental approach depends on rank to provide a valuation framework, it is unsurprising the values associated with the interventions elicited using incremental approach do not offer increased discrimination.

The values associated with rank are strongly discriminatory for the incremental approach. This indicates face validity as it corresponds to the underlying economic theory and provides evidence of the ability of the incremental approach to extrapolate value, which theoretically accurately represents the participant’s consumer surplus. The standard approach provides increasing values from the fourth preference onward, but the intervals are smaller. Assuming participants are not overstating their maximum WTP, this indicates the incremental approach is superior in capturing the additional value of the next preferred option.

The top-ranked intervention in the standard approach, supervised tooth brushing, receives the lowest median value in the standard approach. The incremental approach remains consistent with theory and produces a result that corresponds with the explicit rank. Previous studies and the WTP literature identify the most likely explanation for the discrepancy in the standard approach is that participants will anchor on perceptions of cost.^5^ Participants are likely aware of the costs associated with brushing their own teeth, with the only other additional cost described by the scenario being a nurse to train teachers. This may be thought of as a “cheap” intervention and, as such, may receive a lower value. Fluoride varnish, another intervention that may be perceived of as low cost as it requires few additional resources, receives a similarly low value. These results identify a potential limitation of the cheap talk script as it may have been ineffectual when attempting to eliminate bias from results. However, it is also important to consider these interventions in the wider context of the questionnaire. These services are the only two that would be provided through public health initiatives, which may affect the valuation. These services are also the only ones to specifically target deprived communities, and there may be an element of altruistic signaling.^7^ A participant can be seen as supporting the notion of helping those who are disadvantaged by giving the service a high rank but may be reluctant to support it financially—either generally through paying additional tax or more specifically through the services suggested, as indicated by the protest responses.

## Conclusion

The evidence in this article identifies that the incremental approach to WTP is superior to the standard approach yet cannot conclude that the incremental approach is fully consistent with theory. Further research should be conducted into the issue of protest responses within the incremental approach and how they should be considered in the context of preference reversals. In the interim, those seeking to elicit values for multiple interventions should consider using the incremental approach, particularly in a policy content. The behavior displayed by respondents using protest responses has wider implications for the definition, use, and analysis of zero responses in WTP questionnaires.
